# Systematic re-evaluation of intraoperative motor-evoked potential suppression in scoliosis surgery

**DOI:** 10.1186/s13013-018-0161-3

**Published:** 2018-07-02

**Authors:** Yew Long Lo, Yam Eng Tan, Sitaram Raman, Adeline Teo, Yang Fang Dan, Chang Ming Guo

**Affiliations:** 1 Department of Neurology, National Neuroscience Institute, Singapore General Hospital, Outram Road, Academia Level 4, Singapore, 169608 Singapore; 2 Duke-NUS Medical School, Singapore, Singapore; 3 Singapore General Hospital, Singapore, Singapore

**Keywords:** Intraoperative monitoring, Motor-evoked potential, Suppression, Amplitude, Scoliosis, Anesthesia

## Abstract

**Background:**

Motor- (MEP) and somatosensory-evoked potentials (SSEP) are susceptible to the effects of intraoperative environmental factors.

**Methods:**

Over a 5-year period, 250 patients with adolescent idiopathic scoliosis (AIS) who underwent corrective surgery with IOM were retrospectively analyzed for MEP suppression (MEPS).

**Results:**

Our results show that four distinct groups of MEPS were encountered over the study period. All 12 patients did not sustain any neurological deficits postoperatively. However, comparison of groups 1 and 2 suggests that neither the duration of anesthesia nor speed of surgical or anesthetic intervention were associated with recovery to a level beyond the criteria for MEPS. For group 3, spontaneous MEPS recovery despite the lack of surgical intervention suggests that anesthetic intervention may play a role in this process. However, spontaneous MEPS recovery was also seen in group 4, suggesting that in certain circumstances, both surgical and anesthetic intervention was not required. In addition, neither the duration of time to the first surgical manoeuver nor the duration of surgical manoeuver to MEPS were related to recovery of MEPS. None of the patients had suppression of SSEPs intraoperatively.

**Conclusion:**

This study suggests that in susceptible individuals, MEPS may rarely occur unpredictably, independent of surgical or anesthetic intervention. However, our findings favor anesthetic before surgical intervention as a proposed protocol. Early recognition of MEPS is important to prevent false positives in the course of IOM for spinal surgery.

## Background

Motor-evoked potentials (MEPs) are routinely recorded during intraoperative monitoring (IOM) for spinal surgery to ensure integrity of the descending motor tracts. In addition, somatosensory-evoked potentials (SSEP) monitor the ascending dorsal column pathways of the posterior cord. Noteworthy, patients with scoliosis are neurologically intact in general, compared to those undergoing surgery for intramedullary or extramedullary spinal cord disorders.

MEPs and SSEPs are susceptible to the effects of intraoperative environmental factors. For MEPs, volatile anesthetic agents suppress excitability of the motor cortex, resulting in diminished amplitudes [1]. To mitigate the effects of inhalational anesthetics, total intravenous anesthesia (TIVA) has been shown to be effective [2].

SSEPs, in contrast, are less susceptible [3] to the dose-dependent effects of anesthetic agents compared to MEPs. However, blood pressure, anemia, and temperature appear to influence latency and amplitude of responses [4].

Hence, optimal conditions for IOM of spinal surgery include stable blood pressure and core temperature, avoidance of excessive blood loss, use of TIVA to maintain an adequate depth of anesthesia, as well as awareness of the effects of inhalational or neuromuscular blocking agents.

Gradual suppression of MEPs has been observed in patients under general anesthesia. MacDonald et al. [5] noted abrupt lower limb MEP loss during prolonged scoliosis surgery restored after instrumentation release without deficit and suggested increasing MEP stimulation parameters to offset this effect. Lyon et al. [6] described “anesthetic fade” by virtue of the rate of rise of stimulation voltage threshold proportional to anesthetic duration. The observed effect appears to be more pronounced in myelopathic than neurologically normal patients, but the underlying mechanism responsible remains unclear. In contrast, Holdefer et al. [7] reviewed MEP amplitudes of 50 patients receiving desflurane or propofol during spinal deformity surgery but found no evidence of reducing trend with time. This was corroborated by a separate study [8] which also found no significant MEP amplitude changes over 120 min during propofol anesthesia for spinal surgery. In all, there appears to be conflicting evidence for the occurrence of MEP amplitude reduction intraoperatively and if this phenomenon, if present, is related to anesthetic duration.

We attempt to re-examine these issues relating to motor-evoked potential suppression (MEPS) during IOM in the current study.

## Methods

The institution’s ethics committee had previously approved the study protocols.

Over a 5-year period, 250 patients with adolescent idiopathic scoliosis (AIS) who underwent corrective surgery with IOM were retrospectively reviewed. All were previously determined by a neurologist to be without deficits. IOM recordings whereby MEP amplitude reductions beyond 50% of baseline value at maximum cortical stimulation of 400 V were defined as MEPS and identified for further analysis. Similarly, IOM recordings whereby SSEP amplitude reduction beyond 50% of baseline value identified for further analysis. The IOM protocol using TIVA and cortical stimulation methodology have been published previously [9, 10].

For induction of anesthesia, propofol at 1–2 mg/kg and fentanyl at 2 μg/kg was administered. A single administration of 0.8 mg/kg intravenous atracurium was used to facilitate endotracheal intubation. No further doses of neuromuscular blocking agents were used subsequently. Anesthesia was maintained using the regimen of 10 mg/kg propofol for the first 10 min, 8 mg/kg for the next 10 min, and 6 mg/kg for the subsequent length of operation. Fifty percent air in oxygen was administered. Remifentanil at a dose range of 0.03–0.1 μg/kg/min and morphine were titrated as required for pain relief. Electrocardiography, pulse oximetry, capnography, and direct radial artery pressures were monitored. A bispectral index (BIS) monitor was used in 6 of the patients. All patients were kept normothermic with a warming blanket, and normotensive anesthesia was maintained throughout the operation. Where the BIS monitor was used, the depth of anesthesia was kept to about 40 on the index. As 40 to 60 is considered the range for adequate depth of anesthesia, this was at the deeper end of the range.

After approximately 45 min post-induction, a train of four-twitch assessment was performed using a nerve stimulator (Fischer Paykel NS242, United Kingdom) on the median nerve at the wrist. Cortical stimulation was commenced only when the amplitude of the fourth twitch (abductor pollicis muscle) was visibly similar to the first, suggesting that the effects of neuromuscular blocking agents have subsided.

Cortical stimulation was delivered by 9-mm gold-plated disc electrodes at C3C4 (International 10-20 system) affixed with collodion. C3 was the active stimulating electrode position for left cortical stimulation, while C4 was for right cortical stimulation, correspondingly to a cross scalp stimulating configuration. A train of 5 square wave stimuli 0.5 ms in duration was delivered at 4 ms (250 Hz) interstimulus intervals. Stimulation output was increased in steps of 50 V until a morphologically reproducible MEP with the largest amplitude was elicited. The intensity was then increased and fixed at 10% above this threshold intensity to obtain a supramaximal MEP response recorded with 13-mm disposable subdermal needles (Technomed Europe, Beek, Netherlands) in the tibialis anterior (TA) bilaterally. Amplifier filter settings were set at 10 Hz and 2 kHz. Input impedance of stimulating and recording electrodes was maintained below 5 kΩ.

MEPs from the TA muscles were recorded bilaterally from the lower limbs by means of a Nicolet Endeavor CR IOM system (Natus Technology, USA). Peak to peak amplitudes and onset latency was measured for MEP responses in each limb, obtained from ipsilateral and contralateral cortical stimulation; ipsilateral MEPs refer to MEPs recorded from the TA on the same side as cortical stimulation. Ten consecutive supramaximal MEPs obtained before insertion of pedicle screws were averaged to obtain a final mean amplitude and latency as a baseline. The baseline sensitivity for signal acquisition was kept at 50 μV.

Surgical maneuver refers to screw placement or rod placement (Table 1).

**Table 1:** Summary of MEPS events for 12 patients with AIS

Patient	Age	Sex	Start anesthesia	Surgical maneuver	MEP suppression	Surgical intervention	Anesthetic intervention	MEP recovery	Stage of operation	Clinical outcome
Group 1										
1	17	F	1319	1550	1609	1618	1622	1626	Screw plac	N
2	14	F	0850	0950	1001	1006	1204	1246	Screw plac	N
3	23	M	0959	1308	1346	1721	1730	1800	Nil	N
4	11	F	0845	1058	1101	1108	1150	1214	Screw plac	N
Group 2										
5	13	F	1334	1518	1534	1542	1647	No	Screw plac	N
6	21	F	1240	1416	1423	1426	1530	No	Left rod plac	N
7	29	F	0919	1131	1212	1258	1318	No	Screw plc	N
8	13	F	1411	1522	1535	1545	1600	No	Left rod plac	N
Group 3										
9	21	M	0908	1118	1133	No	1212	1329	Screw plac	N
10	11	F	1025	1041	1154	No	1317	1322	Screw plac	N
11	36	F	1428	1806	1811	No	1819	1849	Screw plac	N
Group 4										
12	31	F	0926	1004	1045	No	No	1112	Screw plac	N

*M* male, *F* female, *Plac* placement, *MEP* motor-evoked potential, *AIS* adolescent idiopathic scoliosis, *N* normal

Surgical intervention consisted of removal of pedicle screws, brackets, and rods, followed by a wake up test. The decision for wake up tests is left to the surgeon ultimately. No wake up tests were performed for cases 9 to 12 as it was felt that there was sufficient recovery of MEPs without surgical intervention.

Anesthetic intervention referred to the temporary reduction of TIVA and remifentanil to allow for the reduction in the depth in anesthesia. In 6 cases where the BIS was used, the value rose to around 60 or more indicating a greater probability of deep sleep rather than anesthesia.

MEP amplitude recovery was defined as the recovery MEP amplitude to beyond 50% of baseline value or latency delay < 10% of baseline value.

SSEP amplitude recovery is similarly defined as the recovery cortical SSEP (P37) amplitude to beyond 50% of baseline value or latency delay < 10% of baseline value.

The following time intervals were noted for each case of possible MEPS:

1. Start of anesthesia to MEPS
2. MEPS to surgical intervention
3. MEPS to anesthetic intervention
4. Start of anesthesia to surgical maneuver
5. Surgical maneuver to MEPS

An anesthetist not involved in the management of each case reviewed intraoperative data to ensure that no confounding factors for MEPS, including vital signs, anesthetic protocol, and interventions, were present.

The Kruskal-Wallis and Mann-Whitney *U* tests were used to compare interval between groups. A *p* value < 0.05 denoted statistical significance.

## Results

A total of 12 AIS patients (2 men; mean age 20 (range 11 to 36)) were included in the final analysis. All were clinically well with no transient or permanent neurological deficits after surgical correction.

Based on 250 patients analyzed in this cohort, the estimated frequency of MEPS is 4.8%.

We identified four groups of cases fulfilling MEPS criteria:

1. MEPS with anesthetic and surgical intervention, followed by MEP amplitude recovery
2. MEPS with anesthetic and surgical intervention, without MEP amplitude recovery
3. MEPS with anesthetic but no surgical intervention, followed by MEP amplitude recovery
4. MEPs without anesthetic and surgical intervention, followed by MEP amplitude recovery

Wake up tests performed for cases 1 to 8 all normal.

In group 3, intraoperative visual monitoring devices such as the O Arm (Medtronic, plc, Colorado, USA) were utilized to provide additional information to the surgical team.

There were no significant differences in time interval 1 between groups 1, 2, and 3 (Kruskal-Wallis test, *H* = 0.712, *p* > 0.05). For time interval 2, comparison between groups 1 and 2 did not reveal statistical significance (Mann-Whitney *U* test, *Z* = 0.104, *p* > 0.05). In addition, no statistical difference between groups 1, 2, and 3 was found for time interval 3 (Kruskal-Wallis test, *H* = 4.348, *p* > 0.05), 4 (Kruskal-Wallis test, *H* = 0.875, *p* > 0.05), and 5 (Kruskal-Wallis test, *H* = 1.095, *p* > 0.05).

Of the 12 cases, all 8 cases with MEP recovery were associated with screw placement. Conversely 2 of 4 cases without MEP recovery (group 2) were associated with rod placement.

The MEP changes of control muscle groups in the upper extremities (first dorsal interossei) did not exceed amplitude reductions beyond 50% of baseline value at maximum cortical stimulation of 400 V.

No intraoperative SSEP changes were detected for all patients.

Tables 1 and 2 summarize clinical data of the 12 cases analyzed.

**Table 2:** Summary of time intervals for 12 patients with AIS

Interval	1	2	3	4	5
Group 1					
1	170	9	13	189	19
2	71	5	123	60	11
3	225	218	224	188	54
4	136	7	49	133	3
Group 2					
1	120	8	73	104	16
2	160	3	67	96	7
3	173	46	66	131	41
4	84	10	25	70	13
Group 3					
1	145		184	130	15
2	89		172	16	13
3	99		261	218	5
Group 4					
1	79			38	41

All values shown are in minutes

## Discussion

Our results show that four distinct groups pf MEPS were encountered over the study period. All 12 patients did not sustain any neurological deficits postoperatively. However, comparing groups 1 and 2 suggest that neither the duration of anesthesia nor speed of surgical or anesthetic intervention were associated with recovery to a level beyond the criteria for MEPS. For group 3, spontaneous MEPS recovery despite the lack of surgical intervention suggests that anesthetic intervention may play a role in this process. However, spontaneous MEPS recovery was also seen in group 4, suggesting that in certain circumstances, both surgical and anesthetic interventions were not required. In addition, comparing groups 1, 2, and 3 suggests that neither the duration of time to the first surgical maneuver nor the duration of surgical manoeuver to MEPS were related to recovery of MEPS. However, it appears that rod placement (group 2) (Table 1) is associated with more closely with failure of MEP recovery. As the numbers involved are small, it would be difficult to make broad conclusions based purely on these observations.

Overall, this study suggests that in susceptible individuals, MEPS may rarely occur unpredictably, independent of surgical or anesthetic intervention, although the observations made were not on an intentional interference basis. The eventual clinical outcomes were, most importantly, favorable. However, anesthetic intervention was performed in 11 of 12 cases and surgical intervention in 8 of 12 cases. Of note, case 4 in group 1 and cases 9, 10, and 11 in group 3 all had anesthetic intervention and MEP amplitude recovery under BIS monitoring. While the exact reasons remain unclear, the results overall favor anesthetic intervention over surgical intervention. In addition, over the study period, there were no identified cases fulfilling the criteria for significant SSEP changes, suggesting that this form of monitoring may be less vulnerable to interference by external factors intraoperatively. This was seen in contrast to previous reports [11, 12].

There is a lack of published information with regard to MEPS. One previous study [6] found the measured voltage threshold needed to produce an MEP of 50 μV to be greater at the end of surgery than at baseline. The rate of rise of this threshold was also greater in relation to operating duration and presence of myelopathy. However, direct comparison to our findings may not be valid in view of differences in stimulating and recording parameters, as well as inclusion of neurologically abnormal patients.

To our knowledge, no further published studies have attempted to address MEPS during IOM systematically in a similar manner.

What can we conclude on the physiological basis of MEPS? Several mechanisms have been proposed previously regarding the effect of anesthetic agents on MEPs. These include actions on synaptic transmission, prolongation of axonal refractory period, depressing spinal motor neuron excitability, and facilitation of GABA-mediated inhibitory interneuron actions [13–15]. Our findings are based on elimination of confounding intraoperative factors of blood loss, hypotension, hypothermia, and a standardized anesthetic intervention strategy in neurologically normal patients. In spite, the results do not suggest a duration-dependent suppression of cortical excitability for MEPS. Rather, individual factors predisposing to desynchronization of descending volleys summating at spinal interneurons or motor neurons may play a role, but this remains to be further explored.

Hence, we prefer the term MEPS over “anesthetic fade” in view of uncertainty over the purported physiological mechanisms, as well as lack of evidence of dose or duration anesthesia plays in AIS patients.

As current studies are limited by small patient numbers, collation of experiences among multiple IOM centers utilizing similar protocols may shed more light on MEPS. Anesthetic depth measurements such as the bispectral index and electroencephalograhy can be incorporated to reduce additional confounders. Monitoring MEPs obtained from an extra muscle group in the lower limb may help mitigate the possibility of recording MEPs with diminished amplitude as a result factor not directly related to neurophysiological dysfunction.

**Fig. 1:**
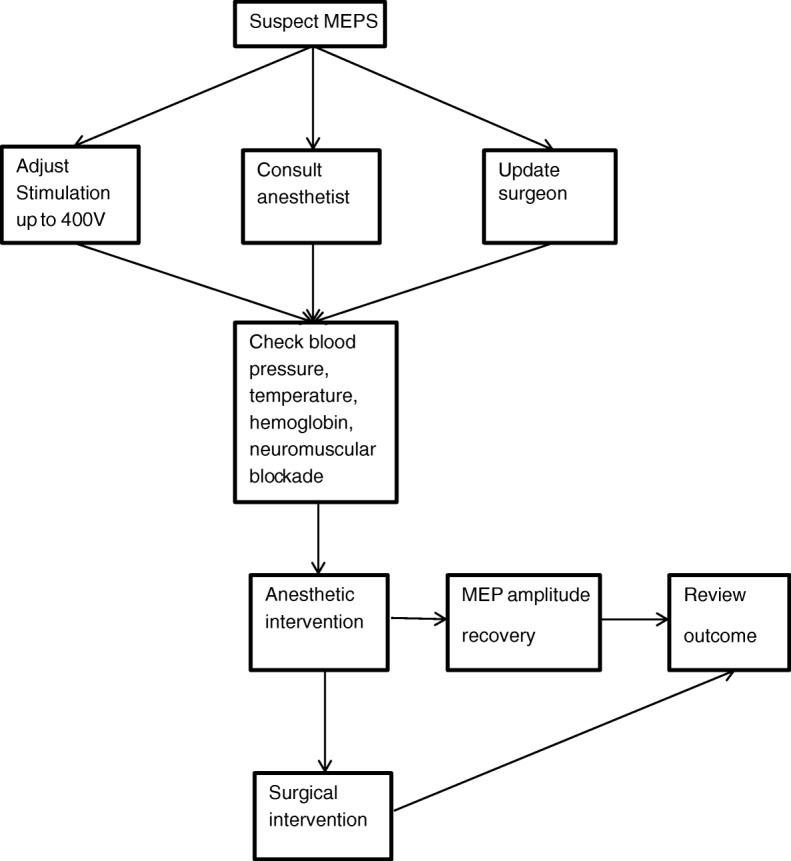
Flow diagram of a suggested protocol of action for MEPS in scoliosis surgery

Figure 1 is a flow diagram of a suggested protocol when MEPS is encountered, highlighting the use of anesthetic before surgical intervention based on findings of the current study. Early recognition is important to prevent false positives in the course of IOM for spinal surgery.

Conversely, a recent study examining 62,038 spine surgeries of all categories retrospectively had determined that false negatives occur at a rate of 0.04% [16]. It would appear that if MEPS occurring at 4.8% contributes to false positives, then the only patient in group 4 could be considered as a “true” false positive, rendering the overall rate to be 0.4%. Our study, however, consisted of only neurologically normal AIS patients instead of all patients undergoing spinal operations.

For IOM using MEPs overall, it is recommended that interpretation should take into consideration limitations, confounding factors, and the MEP warning criteria be tailored to the type of surgery, as well as the technique and experience of the monitoring team [17]. To date, disappearance of the recorded MEP signals is the main warning criterion yet proposed for spinal cord monitoring. This is based on variability that challenges other criteria, high sensitivity to central motor disturbances, the likelihood that pathophysiology will affect many corticospinal axons because the tract is very small in the spinal cord, and the rapid failure of ischemic lower motor neurons [18–20].

## Conclusions

This study suggests that in susceptible individuals, MEPS may rarely occur unpredictably, independent of surgical or anesthetic intervention. However, our findings favor anesthetic before surgical intervention as a proposed protocol. Early recognition of MEPS is important to prevent false positives in the course of IOM for spinal surgery.

## Abbreviations

BIS: Bispectral index
IOM: Intraoperative monitoring
MEP: Motor-evoked potential
MEPS: Motor-evoked potential suppression
SEP: Somatosensory-evoked potential
TA: Tibialis anterior
TIVA: Total intravenous anesthesia
